# Spontaneous Passage of Gallbladder Calculi-Facilitation by Endoscopic Sphincterotomy

**DOI:** 10.1155/1998/63823

**Published:** 1998-08

**Authors:** B. C. Sharma, D. K. Agarwal, S. S. Baijal, V. A. Saraswat

**Affiliations:** 1 Departments of Gastroenterology and Radiology Sanjay Gandhi Postgraduate Institute of Medical Sciences Lucknow India; 2 591, Sector 2 Panchkula India

## Abstract

After Endoscopic sphincterotopy (ES) gallbladder motility increases leading to expulsion of crystals and stones. But this is not a universal phenomenon. We evaluated cholangiographic findings in patients emptying their gallbladder after ES for common bile duct (CBD) stones. Cholangiographic features of twenty patients expelling gallbladder calculi after ES were studied. Controls included 20 age and sex matched patients with gallstones and CBD stones, who did not expel gallstones after ES. Of 20 cases in study group, 9 recovered more than 20 stones each in the stool within 7 days of ES. Repeat ERCP showed empty gallbladder in all, whereas CBD was full of stones in 11 of the 20 cases. In the study group, low insertion of the cystic duct was more common (10 vs 0, *p*<0.04), the cystic duct made a narrow angle (20±5° vs 50±10°, *p*<0.04) with CBD before insertion and cystic duct diameter was higher (5 mm vs 2.5 mm, *p*<0.04) as compared to controls. We conclude that in patients undergoing ES with intact gallbladder and small gallbladder calculi, spontaneous emptying of gallbladder calculi occurs, if cystic duct is wider, has low insertion and makes narrow angle with CBD before insertion.


*HPB Surgery*, 1998, Vol. 11, pp. 23–26
Reprints available directly from the publisher
Photocopying permitted by license only

© 1998 OPA (Overseas Publishers Association) N.V.
Published by license under
the Harwood Academic Publishers imprint,
part of The Gordon and Breach Publishing Group.
Printed in India.

### Spontaneous Passage of Gallbladder Calculi-Facilitation by Endoscopic Sphincterotomy

B. C. SHARMA*, D. K. AGARWAL, S. S. BAIJAL and V. A. SARASWAT

*Departments of Gastroenterology and Radiology, Sanjay Gandhi Postgraduate Institute of Medical Sciences, Lucknow (India)*

*(Received 2 January 1996; In final form 15 May 1997)*

**After Endoscopic sphincterotopy (ES) gallbladder motility increases leading to expulsion of crystals and stones. But this is not a universal phenomenon. We evaluated cholangiographic findings in patients emptying their gallbladder after ES for common bile duct (CBD) stones. Cholangiographic features of twenty patients expelling gallbladder calculi after ES were studied. Controls included 20 age and sex matched patients with gallstones and CBD stones, who did not expel gallstones after ES. Of 20 cases in study group, 9 recovered more than 20 stones each in the stool within 7 days of ES. Repeat ERCP showed empty gallbladder in all, whereas CBD was full of stones in 11 of the 20 cases. In the study group, low insertion of the cystic duct was more common (10 vs 0, $p<0.04$), the cystic duct made a narrow angle ($20\pm5°$ vs $50\pm10°$, $p<0.04$) with CBD before insertion and cystic duct diameter was higher (5 mm vs 2.5 mm, $p<0.04$) as compared to controls. We conclude that in patients undergoing ES with intact gallbladder and small gallbladder calculi, spontaneous emptying of gallbladder calculi occurs, if cystic duct is wider, has low insertion and makes narrow angle with CBD before insertion.**

*Keywords:* Cholelithiasis, choledocholithiasis, cholangitis, papillotomy, cystic duct, cholangiogram

### INTRODUCTION

Endoscopic sphincterotopy (ES) is an established mode of treatment for retained or recurrent stones in the common bile duct after cholecystectomy. With increasing experience, the indications for ES have expanded to include patients with gallstones and common bile duct stones with poor operative risks. In some centres ES is performed in patients with an intact gallbladder, who present with cholestasis, cholangitis and gallstone pancreatitis regardless of surgical risk [2]. It has been reported both in experimental and clinical studies that ablation of the papillary sphincter enhances gallbladder emptying. After ES gallbladder motility increases and expels crystals and stones [3–9]. But this phenomena is not a universal finding. Only in a few patients has it been found on routine check ERCP that, following ES, the gallbladder becomes empty and stones come to lie in the common bile duct. Though gallbladder motility increases after ES in

*Corresponding author: Prestent address: 591, Sector 2, Panchkula, Haryana (India).

all patients, only a few empty their gallbladder and this fact tempted us to make a detailed evaluation of cholangiographic findings in 20 of our patients, exhibiting this phenomena.

### PATIENTS AND METHODS

Of 120 patients who had undergone ES with the gallbladder in situ, 20 patients cleared their gallbladder of stones. Endoscopic procedure was performed using a JFIT 10 or TJF 10 sideviewing duodenoscope ES(1–2 cm) was done using a standard sphincterotome. Dormia extraction was tried in every patient. The check ERCP was done in every patient at an interval of 7 to 30 days, after the first procedure. All the patients were requested to check their stools for stones after ES. Cholangiographic findings were studied in patients expelling calculi from gallbladder after ES with special reference to number and size of gallbladder calculi, common bile duct diameter, number and size of common bile duct calculi, cystic duct diameter, level of cystic duct insertion and angle cystic duct makes with common bile duct before insertion. Measurement of common bile duct and cystic duct were taken at the site of their maximum diameter. Cystic duct was considered to run parallel to common duct, if the course of the two ducts was closely adherent for at least 1 cm. For determining the location of the junction along the length of extrahepatic biliary tree, the distance from ampulla to bifurcation of the duct was divided into three equal parts and expressed a proximal, middle and distal one-third. Cystic duct joining common duct in distal one-third was considered as low insertion of cystic duct [10]. The cholangiographic findings were compared with 20 age and sex matched controls, having multiple small gallstones with common bile duct stones and functioning gallbladder. They had undergone ES for common bile duct stones, had clear common bile duct on check ERCP but did not expel gallbladder stones after ES. In controls also the check ERCP was performed at intervals ranging from 7–30 days.

### RESULTS

Patients included 8 men and 12 women with a mean age of 42 years (range 30 to 60 years). All the patients has cholelithiasis with choledocholithiasis and 6 patients has associated cholangitis. Liver function tests were as shown in Table I. In all of the 20 patients cholangiograms revealed 1 to 5 common duct stones, which were actively extracted after doing ES and the common bile duct was cleared in every patient. Of 20 patients, 9 recovered more than 20 stones in the stool within 7 days of ES. Repeat ERCP done at intervals of 7 to 30 days, showed an empty gallbladder in all, whereas the common bile duct was full of small stones (4 to 6 mm) in 11 out of 20 patients.

On cholangiographic evaluation (Tab. II), the gallbladder was opacified in all the cases and revealed multiple (10 to 35) calculi of 4 to 6 mm.

TABLE I Liver function tests before and after ES, $n = 20$

| | Before ES | After 7 days |
|---|---|---|
| | Mean ± SD (range) | Mean ± SD (range) |
| 1. Total bilirubin (mg/dL) | 5.5 ± 4.4 (0.6–14.3) | 1.1 ± 0.6 (0.4–1.6) |
| 2. Direct bilirubin (mg/dL) | 4.1 ± 4.0 (0.1–12) | 0.3 ± 0.1 (0.2–0.4) |
| 3. Alkaline phashatase (IU/L) | 381.7 ± 281 (132–810) | 126 ± 26 (98–130) |
| 4. AST (IU/L) | 99.2 ± 88.1 (48–290) | 38.6 ± 14.7 (22–50) |
| 5. ALT (IU/L) | 105 ± 87.7 (31–317) | 32.6 ± 11.3 (20–42) |
| 6. Albumin (g/dL) | 3.8 ± 0.4 (3.1–4.5) | 3.2 ± 0.4 (2.9–3.5) |

TABLE II Cholangiographic features

| | Patients $n = 20$ | Controls $n = 20$ | $P$ value |
|---|---|---|---|
| A. Gall bladder calculi | | | |
| Size | 4–6 mm | 4–6 mm | NS |
| Number | multiple | multiple | |
| B. Common bile duct | | | |
| Diameter | 10 mm (7–20 mm) | 10 mm (6–20 mm) | NS |
| Calculi | multiple | multiple | |
| C. Cystic duct | | | |
| Diameter | 5 mm (4–6 mm) | 2.5 mm (2–3 mm) | < 0.04 |
| Insertion | | | |
| Normal | 10 cases | 20 cases | <0.04 |
| Low | 10 cases | 0 | |
| Angle | 20 ± 5° | 50 ± 10° | < 0.04 |

Cystic duct had insertion in lower part of common bile duct in 10 patients. In 6 patients it was running parallel to common bile duct before insertion. In all 20 patients the cystic duct made on acute and narrow angle (20 ± 6°, range 10–30°) with common bile duct before insertion. In controls gallbladder calculi were multiple and varied from 4 to 6 mm in size. Cystic duct insertion was in middle portion of common bile duct in all patients and at insertion cystic duct made angle of 50 ± 10° (range 40–60°) with common bile duct.

### DISCUSSION

ES is an established mode of treatment for choledocholithiasis for last 20 years with complications rate of 8% to 10% and mortality of 1% to 2% [7]. Nowdays ES is widely used for treating common bile duct stones in patients with an intact gallbladder. These are the patients who are elderly, frail and a poor risk for surgery or patients with acute biliary disease and gall stone pancreatitis. The fate of the gallbladder in such patients depends on whether the gallbladder harbors calculi or not. The incidence of acute cholecystitis in patients with acalculous gallbladder is 0 to 1% even after long term followup for 7 to 9 years [8]. In patients with gallstones, the incidence of acute cholecystitis is reported to be 16%[9]. After ES in patients with gallbladder in situ, biliary symptoms occur more frequently in patients with gallbladder calculi as compared to patients with acalculous gallbladder [11]. In earlier studies, there are scattered case reports, where gallbladder calculi are cleared after ES [4, 3, 6]. This is an important finding as the gallbladder in such cases will behave like those with acalculous gallbladder in term of developing acute cholecystitis. Such patients with acalculous gallbladder may not require routine cholecystectomy after ES as recommended by some workers. This phenomenon of facilitation of spontaneous passage of gallbladder calculi can be explained by enhanced gallbladder emptying after ES. Hutton etal studied spontaneous passage of glass beads from gallbladder after ES in dogs [3]. One month after bead implantation, dogs with intact sphincter passed 52%, 26%, 22%, 10%, 0% and 0% of beads with diameters of 2, 3, 4, 5, 6 and 8 mm respectively. For the same respective bead diameters, dogs with sphincterotomy passed 90%, 90%, 88%, 75%, 75% and 42% of beads. In a separate set of dogs, cholescintigraphic study performed after sphincterotomy revealed increased gallbladder ejection fraction from 0.46 to 0.76. In addition sphincterotomy also lowered resting and cholecystokinin stimulated gallbladder volume. Similar results have been reported in other experimental studies. In human studies, ES was found to lower resting gallbladder volume and caerulin stimulated residual gallbladder volume. ES also significantly increased ejection fraction and rate constant for gallbladder emptying [12, 13].

Spontaneous passage of gallbladder calculi after ES occurs only in some patients, so apart from alteration in mechanical functions of gallbladder after ES, others factors: cystic duct length, diameter and insertion and common bile duct diameter are thought to be important. After critical evaluation of cholangiographic findings, we could identify certain distinct features. In all the patients when expelled their the gallbladder has multiple small stones, broader cystic duct, low insertion of cystic duct and the cystic duct made narrow angle with common bile duct in all the cases whereas in normal cases it was found to vary from 40° to 60°.

Based on these findings we consider that apart from enhanced gallbladder motility normal cholangiographic variations to also play a role in determining whether patients will expel gall bladder calculi after ES or not. However a prospective larger study is needed to draw further definite conclusions. In patients undergoing ES with intact gallbladder and small sized gallbladder claculi, if these cholangiographic features are present, the chances of spontaneous passage of gallbladder calculi are there. Unless there is a pressing need such cases can be followed up the with hope of spontaneous emptying of gallbladder. However if stones are large and cystic duct diameter is small then cystic duct occlusion may occur leading to complications like mucocoele and empyema.

### *References*

[1] Simon, M. D., Brooks, W. S. and Herh, T. (1989). Endoscopic sphincterotomy: A reappraisal. *Am. J. Gastroenterol.*, **84**, 213–219.

[2] Siegel, J. H., Safrany, L., Ben-Zvi, J. S. *et al.* (1988). Duodenoscopic sphincterotomy in patients with gallbladder in situ. Report of series of 1272 patients. *Am. J. Gastroenterol*, **83**, 1125–1258.

[3] Hutton, S. W., Sievert, C. E., Vennes, J. A., Shafer, R. B. and Duane, W. C. (1988). Spontaneous passage of glass beads from the canine gall bladders. Facilitation by sphincterotomy. *Gastroenterology*, **94**, 1031–1035.

[4] Rosseland, A. R. and Solhaug, J. H. (1988). Primary endoscopic papillotomy in patients with stones in common bile duct and the gallbladder in situ. A 5–8 years follow up study. *World J. Surg.*, **12**, 111–116.

[5] Moss, J. G., Saunders, J. H. and Wild, S. R. (1985). Endoscopic papillotomy for removal of common bile duct stones without cholecystectomy. *J. Royal Coll Surg Edinburgh*, **30**, 112–114.

[6] Cotton, P. B. and Vallon, A. G. (1982). Duodenoscopic sphincterotomy for removal of bile duct stones in patients with gallbladders. *Surgery*, **91**, 628–630.

[7] Cotton, P. B. (1984). Endoscopic management of bile duct stones (apples and oranges). *Gut*, **25**, 587–597.

[8] Targarona, E. M. and Trias, I. P. M. (1995). Effects of sphincterotomy on gallbladder physiology. *A review. Endoscopy*, **27**, 388–91.

[9] Tanaka, M., Ikeda, S., Yoshimato, H. and Matsumoto, S. (1987). The long term fate of gallbladder after endoscopic sphincterotomy. Complete follow-up study of 122 patients. *Ann. Surg.*, **154**, 505–9.

[10] Shaw, J. M., Dorsher, J. P. and Vennes, J. A. (1993). Cystic duct anatomy: an endoscopic perspective. *Am. J. Gastroenterol.*, **88**, 2102–6.

[11] Hammarstrom, L. E., Halmin, T. and Stridbeck, H. (1996). Endoscopic treatment of bile duct calculi in patients with gallbladder in situ. Long term outcome and factors predictive of recurrent symptoms. *Scand. J. Gastroenterol.*, **31**, 294–301.

[12] Agarwal, D. K., Sharma, B. C., Dhiman, R. K., Baijal, S. S., Negi, T. S., Choudhuri, G. and Saraswat, V. A. (1997). Effect of endoscopic sphincterotomy on gallbladder motility. *Dig. Dis. Sci.*, **42**, 1495–1500.

[13] Sharma, B. C. and Singh, K. (1997). Endoscopic sphincterotomy and gallbladder functions. *Scand. J. Gastroenterol.*, **32**, 95–6.

